# Investigating the Potential Effects of 6PPDQ on Prostate Cancer Through Network Toxicology and Molecular Docking

**DOI:** 10.3390/toxics12120891

**Published:** 2024-12-08

**Authors:** Yuanzhi Song, Wuhong Weng, Shengde Wu

**Affiliations:** 1 Department of Urology, Children’s Hospital of Chongqing Medical University, National Clinical Research Center for Child Health and Disorders, Ministry of Education Key Laboratory of Child Development and Disorders, Chongqing 400014, China; s_yuanzhi@163.com; 2 China International Science and Technology Cooperation Base of Child Development and Critical Disorders, Chongqing 400014, China; 3 Chongqing Key Laboratory of Structural BirthDefect and Reconstruction, Chongqing 400014, China; 4 The First Clinic College, Chongqing Medical University, Chongqing 401331, China; 2019210061@stu.cqmu.edu.cn

**Keywords:** 6PPDQ, prostate cancer, network toxicology, molecular docking

## Abstract

(1) Background: N-(1,3-Dimethylbutyl)-N′-phenyl-p-phenylenediamine-quinone (6PPDQ), as a newly discovered environmental toxin, has been found more frequently in our living conditions. The literature reports that damage to the reproductive and cardiovascular system is associated with exposure to 6PPDQ. However, the relationship between 6PPDQ and cancer still requires more investigation. This research aims to investigate the association between 6PPDQ and prostate cancer. (2) Methods and Results: Based on the data retrieved from the Pharmmapper, CTD, SEA, SwissTargetPrediction, GeneCard, and OMIM databases, we summarized 239 potential targets utilizing the Venn tool. Through the STRING network database and Cytoscape software, we constructed a PPI network and confirmed ten core targets, including IGF1R, PIK3R1, PTPN11, EGFR, SRC, GRB2, JAK2, SOS1, KDR, and IRS1. We identified the potential pathways through which 6PPDQ acts on these core targets using Gene Ontology (GO) and Kyoto Encyclopedia of Genes and Genomes (KEGG) analyses. Ultimately, through molecular docking methods, 6PPDQ binds closely with these ten core targets. These findings indicate that 6PPDQ may influence the proteins related to prostate cancer and may be linked to prostate cancer via several known signaling pathways. (3) Conclusions: This article employs innovative network toxicology to elucidate the prostate carcinogenic effects of 6PPDQ through its modulation of specific vital genes and signaling pathways, thereby establishing a foundational platform for future investigations into the impact of 6PPDQ on prostate cancer and potentially other tumors.

## 1. Introduction

Recently, increasing attention has been devoted to the effects of toxicants in the environment on human health. 6PPDQ is one newly defined chemical substance derived from 6PPD, and it is often added to rubber products to avoid oxidation [1]. Reports indicate that 6PPDQ is detected more frequently in the atmosphere, urban waterways, and soil [2,3].

A recent study reported that 6PPDQ has significantly influenced embryonic zebrafish’s activation and heart rate, resulting in malformation [4]. In another example of animal research, Yao et al. determined that chronic exposure to 6PPDQ had a detrimental effect on semen quality and testosterone levels and yielded significant damage to the structure of the testis and epididymis [5]. At the cell biology level, the toxicity of 6PPDQ has also been demonstrated. For instance, Zhang et al. reported that different concentrations of 6PPDQ altered the metabolism of human liver microsomes [6]. 6PPDQ has been found to change the morphology of differentiated H9c2 cardiomyocytes and disrupt the expression of cardiac-specific markers, as well as support the toxicity of 6PPDQ at the cell level [7].

Furthermore, research on *Homo sapiens* has shown that 6PPDQ and its two primary metabolites are detectable in urine [8], which validates its potential risk to health under exposure to this chemical substance. However, in a particular population, 6PPDQ may enhance this risk. The concentration of 6PPDQ in the cerebrospinal fluid of Parkinson’s patients is twice as much as usual, which is related to the progression of Parkinson’s disease [9]. Furthermore, a lower BMI and increasing morbidity of fever and diarrhea have been found relevant to greater intakes of 6PPDQ in children [10]. Despite these findings, there are few studies regarding the distribution of 6PPDQ in the human body and its potential effects on human health, dramatically emphasizing that more research should be devoted to how 6PPDQ influences the environment and human health. Up till now, the underlying toxicity of 6PPDQ in the liver, heart, reproductive system, etc., has been reviewed [11]. There is a lack of research on the relation between cancer and exposure to 6PPDQ. However, previous studies have reported that some men working in rubber production face an increased risk of prostate cancer [12,13]. However, whether 6PPDQ affects the development of prostate cancer has aroused our doubts.

Network toxicology is new research method based on networks, including bioinformatics, systems biology, and toxicology. This method explores the complex relationship between poisons and diseases and how toxicity emerges with the prediction of potential targets of known substances through utilizing some big data analytics platforms [14]. Many studies have reported using this method to research various toxicants. For example, Chen et al. showed the potential targets of and the mechanism of gastric cancer that is triggered by aspartame [15]. Huang et al. predicted that the core protein and signaling pathway of prostate injury is induced by 4,4′-sulfonyldiphenol [16].

This research probed the possible relationship between 6PPDQ and prostate cancer occurrence, and, through combining the network toxicology method and molecular docking strategies, supporting scientific evidence of the toxicity of 6PPDQ was found, thus driving people’s attention to its latent effects on the environment and human health in the future.

## 2. Materials and Methods

### 2.1. Predicted Target of 6PPDQ

The Canonical SMILES and standard structure of 6PPDQ (CID, 2024, 154926030) were retrieved from the PubChem database (https://pubchem.ncbi.nlm.nih.gov, accessed on 15 July 2024). Potential targets of 6PPDQ were predicted by inputting Canonical SMILES into databases containing Pharmmapper (http://lilab-ecust.cn/pharmmapper/index.html, accessed on 15 July 2024) [17], CTD (https://ctdbase.org, accessed on 15 July 2024) [18], SEA (https://sea.bkslab.org, accessed on 15 July 2024) [19], and SwissTargetPrediction (http://swisstargetprediction.ch, accessed on 15 July 2024) [20], and it was limited to the species of “homo-sapiens” while confirming the search results by comparing the consistency of the structure. We standardized the gene symbols utilizing Uniprot (https://www.uniprot.org, accessed on 15 July 2024) and removed duplicates of the standard target names. Disposal data were kept in Excel. We retrieved them on 10 July 2024.

### 2.2. Collection of the Gene Targets of Prostate Cancer

The related targets of prostate cancer were searched in the GeneCard (https://www.genecards.org, accessed on 15 July 2024) [21] and OMIM (https://www.omim.org, accessed on 15 July 2024) [22] databases with the keyword “prostate cancer”. The data from GeneCard were filtered if the *p*-value was greater than the average. After removing the duplicates, we utilized Venn diagrams to summarize the common targets between 6PPDQ and prostate cancer, considering the common targets as potential parts of 6PPDQ.

### 2.3. Construction of PPI Network and Filtration of Core Targets

Common targets were inputted into the STRING network database (https://cn.string-db.org, accessed on 14 August 2024) [23,24], with limited species “homo-sapiens” only to research potential protein links. “Highest confidence (0.900)” was set as the minimum interactive score, and any disconnected nodes in the network were hidden. Then, the result was imported into Cytoscape (v3.10.1) to construct a PPI network. Core targets were confirmed according to the cluster with the maximum score through utilizing the MCODE plugin [25,26]. The minimum number of degree cutoff was set as 2 for network scoring. For cluster finding, we set the node score cutoff as 0.2, K-Core as 2, and Max. Depth as 100, and we removed singly connected nodes from clusters. With the help of the MOCDE plugin, we obtained some subnetworks ranked by score. The highest-level subnetwork was regarded as the core network, and the genes contained were considered core genes.

### 2.4. Gene Ontology and Pathway Enrichment Analysis

To explore the relevance of overlapping targets between 6PPDQ and prostate cancer, we imported these targets into the DAVID database to gather relevant information for Gene Ontology (GO) and Kyoto Encyclopedia of Genes and Genomes (KEGG) pathway enrichment analyses [27,28]. We conducted a comprehensive analysis of biological processes (BPs), cellular components (CCs), and molecular functions (MFs) to identify the biological function of related targets. The significant pathways of prostate cancer triggered by 6PPDQ were identified through KEGG enrichment analysis. And the top pathways were displayed utilizing the Wei Sheng Xin tool (https://www.bioinformatics.com.cn, accessed on 14 August 2024) based on ranked entries with *p*-value.

### 2.5. Molecular Docking

**Figure 1:**
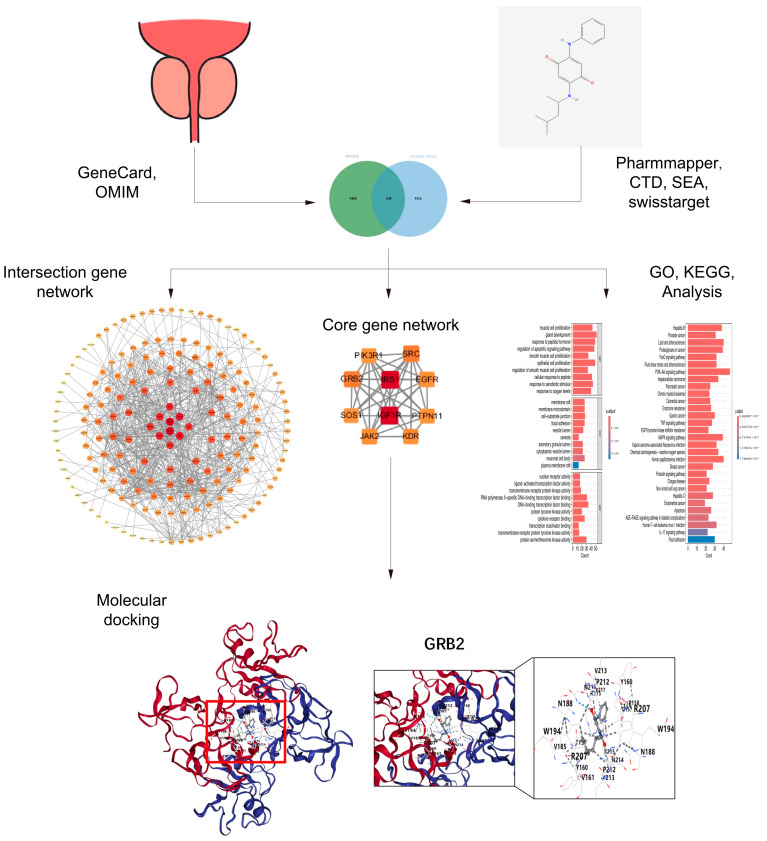
The flow chart of this research.

We evaluate the mechanism and strength of action through molecular docking [29,30]. CB-Dock2 is a newly developed molecular docking technique that performs highly automatic protein–ligand blind docking by four steps: (i) data input, (ii) data processing, (iii) cavity detection and docking, and (iv) visualization and analysis [31,32]. This technology automatically recognizes and combines the unique docking situation of the protein. We retrieved the chemical structure of the 6PPDQ molecule from the PubChem database (https://pubchem.ncbi.nlm.nih.gov, accessed on 15 July 2024), and we searched the PDB structure of various potential targets from the RCSB database (https://www.rcsb.org, accessed on 14 August 2024). Subsequently, all gathered data were uploaded to the CB-Dock2 website to molecular dock. The system of CB-Dock2 displays the initial docking results, and we selected the top one, which was ranked by Vina score. The whole flow is shown as follows (Figure 1).

## 3. Results

### 3.1. Predicted Targets of Prostate Cancer Triggered by 6PPDQ

**Figure 2:**
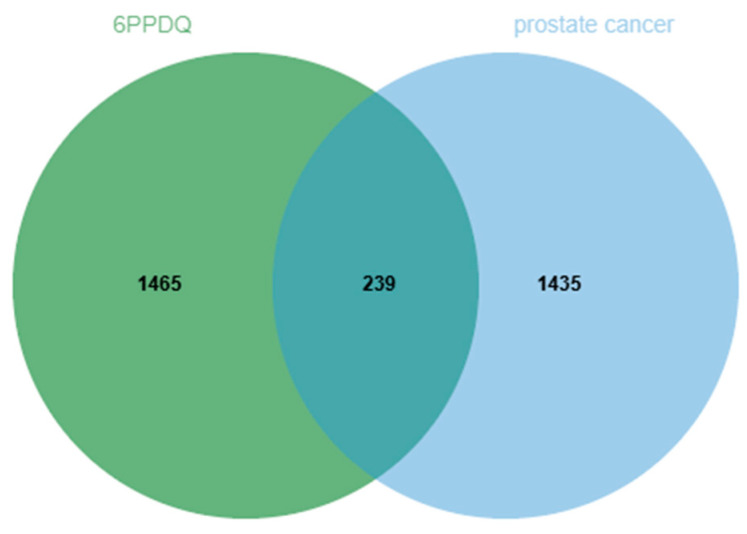
Venn diagram of the overlapping targets of 6PPDQ and prostate cancer. The number of 239 represents the overlapping targets.

After collecting and removing duplicates, 1704 results from the Pharmmapper, CTD, SwissTargetPrediction, and SEA databases were deemed potential targets. Meanwhile, we confirmed 1674 prostate-cancer-related targets from the GeneCard and OMIM databases with filtration. Of these, 239 overlapping targets were revealed using the Venn tool and displayed as follows (Figure 2).

### 3.2. Construction of PPI Network and Identification of Core Target

**Figure 3:**
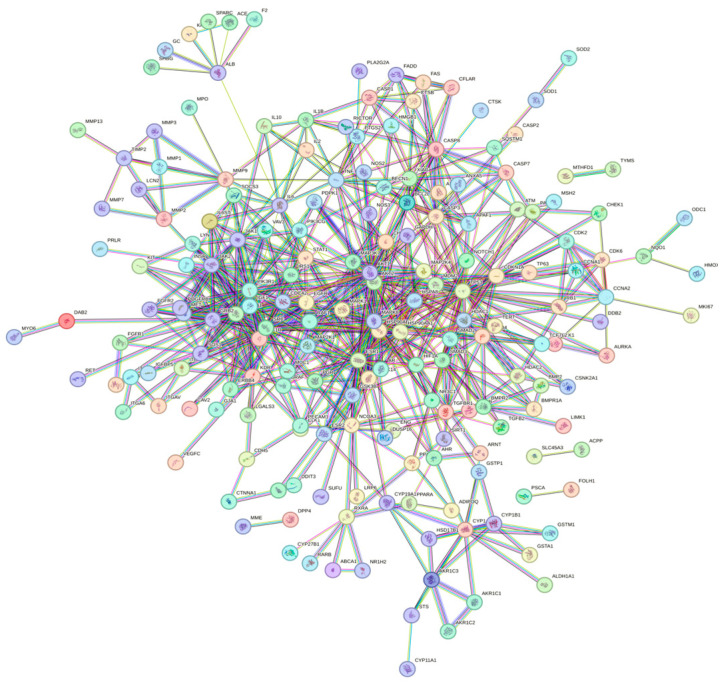
PPI network of common targets generated by STRING.

**Figure 4:**
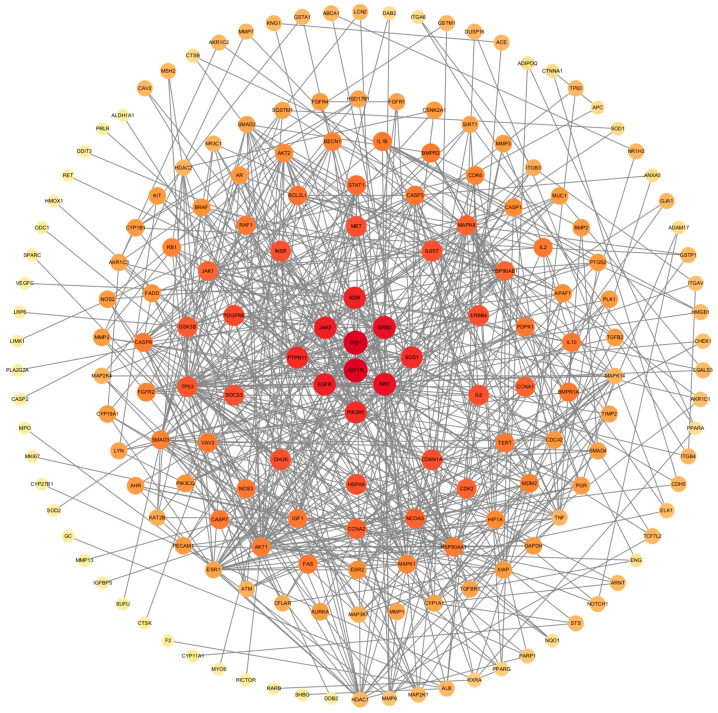
The network of potential targets Each node represents a gene, while the edges indicate their interactions. The size of the node is directly related to its degree, and the intensity of the color reflects the betweenness centrality of the nodes.

**Figure 5:**
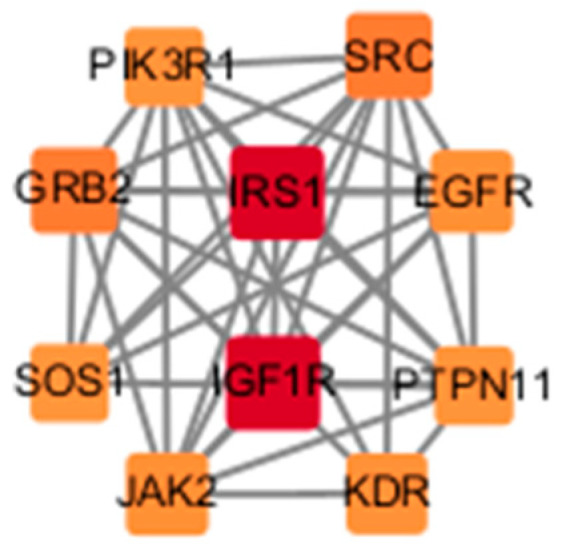
The PPI network of the core targets. Each node represents a gene, while the edges indicate their interactions.

Uploading the data into the STRING database, we constructed a PPI network (Figure 3) with a confidence level of 0.900 with 238 nodes and 657 edges. We performed visualization of this network using Cytoscape software (Figure 4). We ultimately obtained a total of seven subnetworks, with the subnetwork with the highest score attaining 8.667 and encompassing ten genes. The relation of these core targets is displayed as follows (Figure 5, Table 1).

### 3.3. Function of Target Analysis and Biological Pathway Analysis

We analyzed Gene Ontology (GO) and Kyoto Encyclopedia of Genes and Genomes (KEGG) on 239 potential targets. Then, 908 biological processes, 111 cellular components, and 213 molecular functions were determined by utilizing the DAVID database. We ranked the results of the analysis of GO and KEGG by *p*-value. The enriched BP terms were predominantly related to muscle cell proliferation, gland development, and response to peptide hormone. Of ranked CC terms, membrane raft, membrane microdomain, and cell−substrate junction were the main.

**Figure 6:**
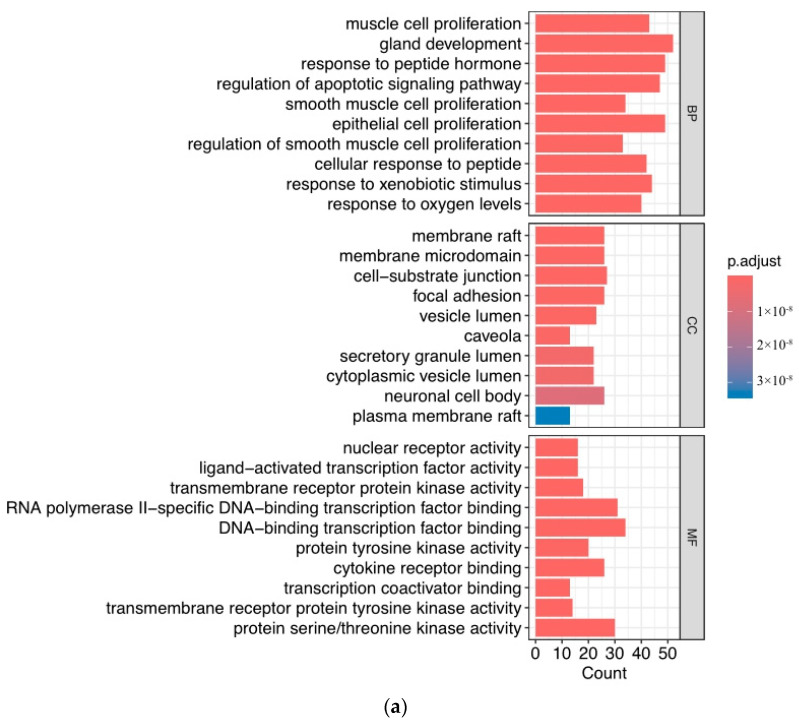

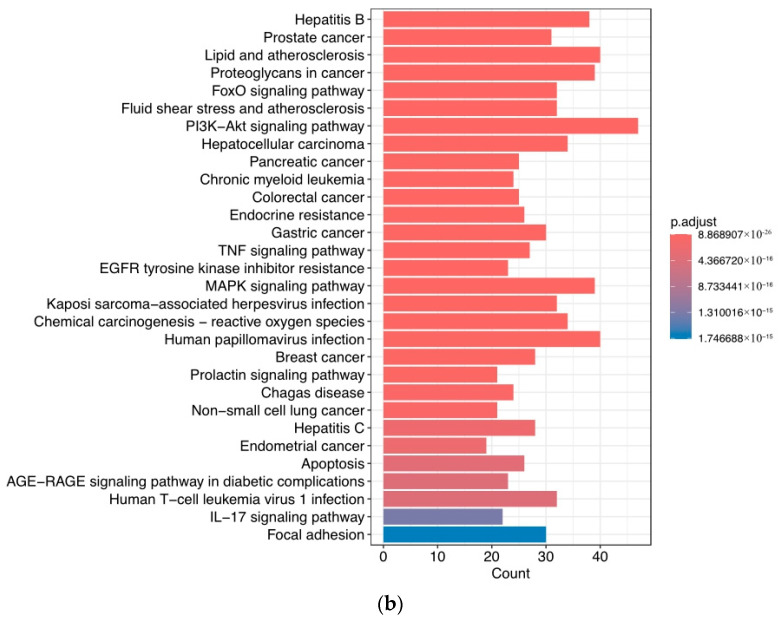
The top 10 GO terms (**a**) and enriched pathways (**b**) of core genes ranked by *p*-value.

Furthermore, the MF terms were enriched in nuclear receptor activity, ligand-activated transcription factor activity, and transmembrane receptor protein kinase activity. The top 10 signaling pathways are ranked (Figure 6a), and lower adjusted *p*-values are colored red, indicating higher enrichment. The ranked enriched signaling pathways were displayed, and the pathway of prostate cancer was situated in the second (Figure 6b).

### 3.4. Results of Molecular Docking

**Figure 7:**
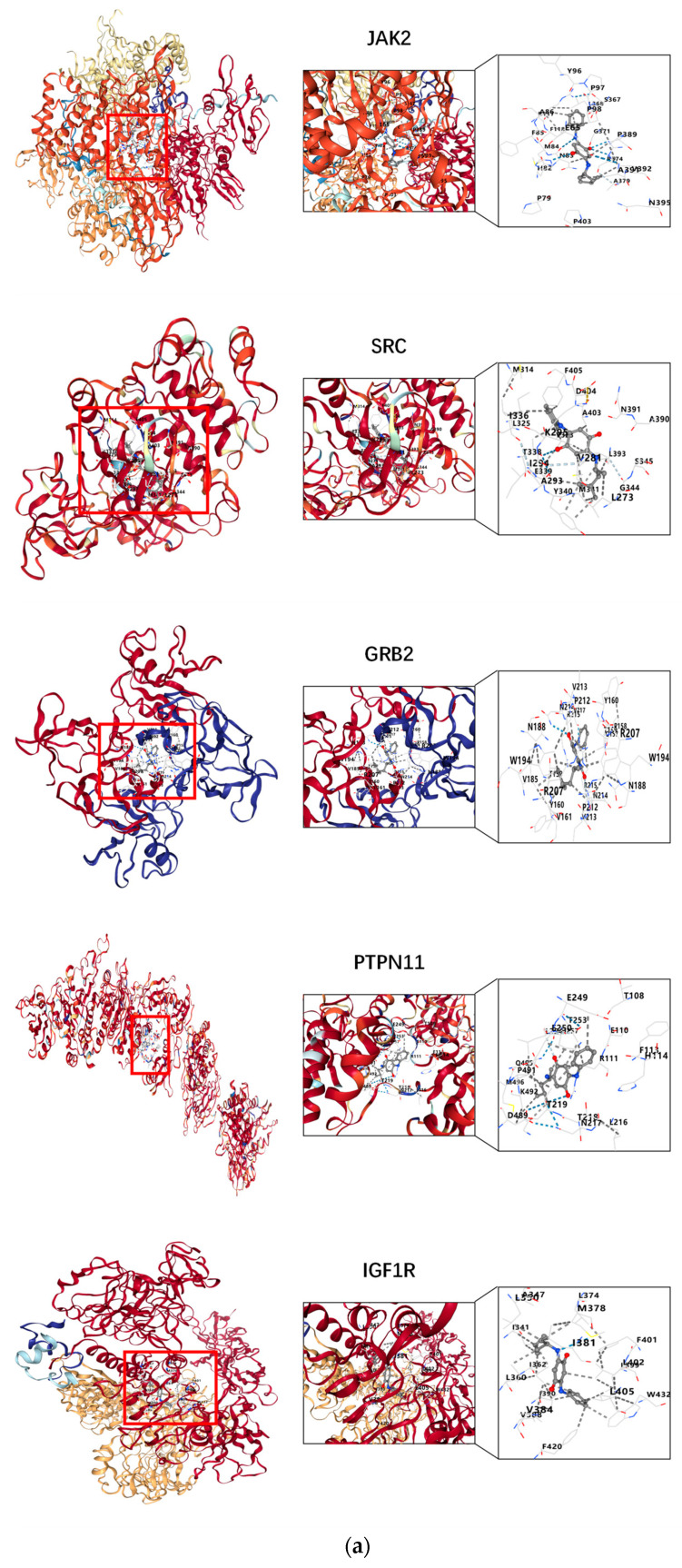

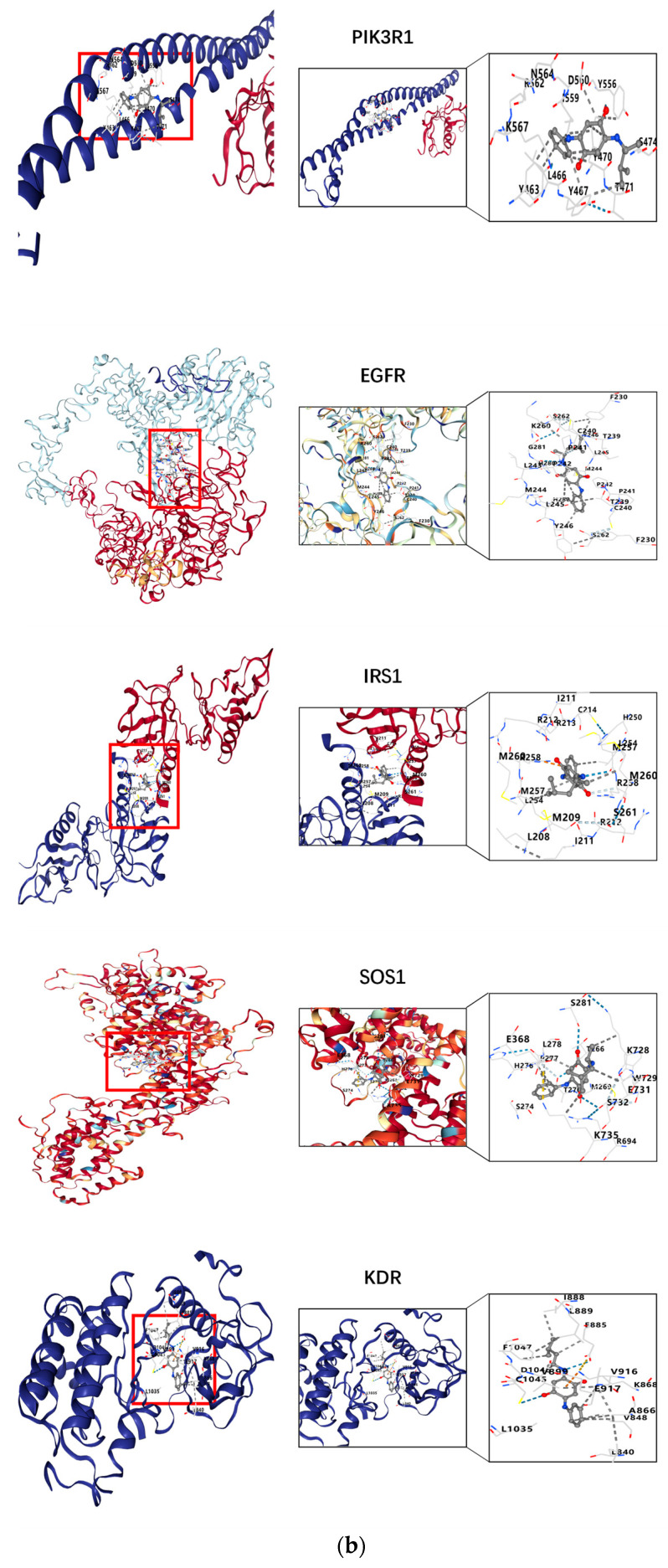
(**a**,**b**) Molecular docking structures with each core target in the lowest Vina score.

The docking interactions between 6PPDQ and the proteins IGF1R, PIK3R1, PTPN11, EGFR, SRC, GRB2, JAK2, SOS1, KDR, and IRS1 were comprehensively analyzed. The structures of the core target proteins docked with 6PPDQ are displayed (Figure 7), while additional information is presented (Table 2, Supplementary Files). According to the Vina score system, a Vina score of less than −5.0 kcal/mol indicates good intermolecular interaction and a lower Vina score suggests better intermolecular affinity. Specifically, a Vina score of less than −7.0 kcal/mol is indicative of stronger binding activity. Our findings reveal that the Vina binding score between 6PPDQ and JAK2 is −8.8 kcal/mol, which is below the threshold of −7.0 kcal/mol. Moreover, it indicates that JAK2 may serve as a vital target regulating the effects of prostate cancer by binding with 6PPDQ. Our analysis indicates that 6PPDQ exhibits a strong affinity for these core proteins, thereby confirming the validity of the preliminary screening results.

## 4. Discussion

### 4.1. Network Toxicology and 6PPDQ

This research employed a network model to analyze predicted targets of 6PPDQ in relation to prostate cancer, drawing data from various databases. By utilizing the STRING network database and Cytoscape software to construct a protein–protein interaction (PPI) network, we identified ten core genes associated with prostate cancer. Additionally, through GO and KEGG analyses, in conjunction with molecular docking strategies, we elucidated the underlying relationship between 6PPDQ and prostate cancer. The network model was systematically applied for pharmacological predictions concerning both known and novel substances [33]. Network toxicology performs toxicity prediction based on this framework for known substances, thereby transforming the conventional paradigm of one drug targeting one disease and potentially leading to significant insights regarding unidentified toxins in the future.

Recent advancements in the field of network toxicology have gained prominence. For instance, network toxicology was used to evaluate the effects of aspartame, which is a kind of controversial food additive, on the occurrence and progress of gastric cancer [15]. Meanwhile, the contribution of bisphenol A to cardiovascular disease was assessed through network toxicology [34]. In addition, the toxicity of the agricultural chemical Thiabendazole has been investigated through this approach [35]. Network toxicology has an advantage in the scientific prediction of toxicity, without numerous advanced studies raising awareness of the potential harm of a new substance and providing direction for future research. One such newly identified substance, 6PPDQ, has been frequently detected in various environmental contexts [2]. It has been proven that 6PPDQ has embryotoxicity and poses risks to reproductive and cardiovascular health [4,5]. Furthermore, detection in the human body is evidence of its possible toxicity, underscoring the necessity for further investigation [8].

This study focuses on a newly defined substance with prostate cancer, which is currently a significant social hazard, to contribute to toxicity research and in-depth understanding of this new substance, elevate public awareness, and establish a foundational basis for the prevention of and reduction in 6PPDQ toxicity in the future.

### 4.2. Core Genes and Prostate Cancer

The identified core genes are highly associated with prostate cancer. IGF1R plays a crucial role in facilitating cell growth and regulating apoptosis and substantially influences tumor development [36]. Elevated levels of IGF1R expression are known to facilitate tumor metastasis [37]. However, many studies reported that aberrant expression of IGF1R correlates with tumor development. For example, NEAT1 will facilitate prostate cancer growth by activating the IGF1R/ATK signaling pathway [38].

Moreover, the abnormal expression of IGF1R out of regulation will contribute to the invasion of prostate cancer. Research conducted by Ozel Capik et al. demonstrated that ectopic overexpression of the CASC11 gene promotes the aggressiveness of prostate cancer cells through the miR-145/IGF1R axis [39]. At the same time, aberrant activation of the PCAT6/IGF2BP2/IGF1R axis will promote both the growth of prostate cancer and its metastasis to bone [40]. PIK3R1 is regarded as a tumor suppressor gene, and increasing evidence suggests that its abnormal expression is linked to heightened cell proliferation and invasion within cancerous tissues [41]. In most types of cancer, PIK3R1 is in a low expression state [42]. It is likely that this gene is in low expression in prostate cancer and is regulated by the environment [43]. Coded by PTPN11, the SHP2 is extensively expressed in human cells and is involved in numerous regulatory processes related to invasion, metastasis, apoptosis, differentiation, and proliferation [44]. After phosphorylation, this protein plays an essential role in AR stabilization, and the increase and decrease in SHP2 activity will increase the probability of malignant tumors [45]. EGFR is of significance to the progression of prostate cancer, exhibiting high expression levels in certain prostate cancer cells [46]. It helps prostate cancer cells survive and keeps them active, metastasizing them to bone [46,47].

Furthermore, it has been proven to possibly be associated with a low survival rate [48]. SRC is a crucial signaling molecule in the process of tumor survival and invasion progress, essential in the regulation and enhancement of cellular functions. SRC has been demonstrated to have significant synergies with the AR, which offers a strong driving force for malignant tumor progression [49,50]. The activation of SRC will contribute to the development of primitive and metastatic prostate cancer [51,52]. Grb2 protein is in the monomer and dimer conversion process, activating and inhibiting MAP kinase, respectively, controlling cancer progression [53]. In addition, Grb2 functions as a molecular signal participating in regulating cholesterol synthesis and the p53 signaling pathway, and the Grb2 gene is highly related to the aberrant activation of proliferation-cycle genes and metabolism-related enzyme genes, especially in the progression of prostate cancer [54]. JAK2 regulates the cell cycle, controls the expression of tumor invasion proteins and anti-apoptotic genes, and plays a vital role in tumor growth, survival, and metastasis [55]. It has been reported that the atypical activation of the JAK2/STAT3 signaling pathway is an essential basis of prostate cancer [56,57]. And this signal routine may promote prostate cancer metastasis by inducing stem-like cell properties [58]. The enhanced expression of SOS1 has been identified as a condition conducive to the proliferation of prostate cancer cell proliferation [59]. Inhibition the high expression of SOS1 has demonstrated therapeutic potential in the treatment of prostate cancer [60]. The overactivation of KDR (VEGFR2) signaling is positively correlated with the deterioration and metastasis of various tumors [61]. In the study of prostate cancer and this gene, it was found that KDR has a specific promoting effect on the proliferation of prostate cancer [62]. IRS1 is closely related to cellular survival, growth, differentiation, and metabolism [63]. The previous literature has reported that the inhibition of IRS1 can suppress prostate cancer proliferation [64]. On the other hand, the abnormal activation of IRS1 promotes prostate cancer progression through the IRS1/SREBP-1 axis [65].

### 4.3. Analysis of GO and KEGG Related to Prostate Cancer

Based on our analysis of the targets in Gene Ontology (GO) and the Kyoto Encyclopedia of Genes and Genomes (KEGG), we discovered that these genes are broadly expressed in subcellular structures and contribute to the development of prostate cancer through various mechanisms. In addition to the direct pathways implicated in prostate cancer, both the FOXO signaling pathway and proteoglycans, as identified in the KEGG pathway analysis, are integral to the disease’s progression. Notably, the FOXO signaling pathway, in particular, is vital for tumor proliferation and growth [66]. As a member of the FOXO subfamily, FOXO3a is involved in regulating various cellular processes, including proliferation and apoptosis, exhibiting a potent inhibitory effect on tumors [67].

The complexity and heterogeneity of proteoglycans allow them to engage in a variety of cellular processes. Specifically, decorin and lumican have shown inhibitory effects on prostate cancer progression, whereas the basement membrane proteoglycan perlecan has been identified as a tumor promoter [68].

Differentiated from mesenchymal cells, smooth muscle cells are the fundamental components of prostate mesenchymal, which can contribute to the inflammatory process by regulating the interaction between the epithelium and stroma [69]. As we all know, the reactive stroma, which contains biological responses and processes similar to those found in granulation tissue during wound healing, is believed to promote the progression of prostate cancer [70]. The reactive stroma initiates in the early stage of prostate cancer and adjusts with the development of cancer, supporting the prostate cancer tissue [71]. However, LNCaP prostate carcinoma cells are essentially nontumorigenic, which can be influenced by variations in stromal composition [72]. In addition, the role of androgen receptors in smooth muscle may impact prostate development by the modulation of IGF-1 signaling pathways [73]. The proliferation of muscle cells may influence the incidence and progression of prostate cancer by altering the composition of the reactive stroma and regulating associated signaling pathways.

The biological processes of “the gland development” and “response to peptide hormones” are highly relevant to prostate cancer. In abundant epidemiological studies, the enhanced risk of mortality was found to be relative to benign prostatic hyperplasia. David D. Ørsted et al. emphasized that benign prostatic hyperplasia (BPH) plays a vital role in predicting prostate cancer progress [74]. As we all know, the average growth of the prostate is regulated by various biological pathways. However, many genes expressed during average prostate growth were found to be re-expressed in cancerous cells, which underlines the stable pathways that maintain a balance between benign hyperplasia and malignant transformation [75,76]. 6PPDQ may influence the prostatic hyperplasia process by affecting these pathways, leading to cancer.

Meanwhile, peptide hormones play a crucial role in prostate carcinogenesis. The development of prostate cancer in the early stage depends on serum androgen levels. Usually, androgen is kept at a stable level under GnRH regulation. However, GnRH was proven to be functional in anti-cancer in autocrine circles, mediating the regulation of pituitary–gonadal axis function [77]. Angiotensin 1–7 was considered to perform anti-proliferative and anti-angiogenic effects in carcinogenic processes, in addition to its cardiovascular effects [78]. In prostate cancer, this hormone can alter the expression of the estrogen receptor gene and even enhance the levels of androgen receptors of prostaglandin-sensitive cells. The complex network of angiotensin influences the aggressiveness of cells [78]. Additionally, animal studies have demonstrated that growth hormones can promote the proliferation and metastasis of prostate cancer cells while affecting the expression of tumor-related genes [79]. Similarly, AZGP1, as with other kinds of secreted proteins, was also verified to be involved in the process of the proliferation and metastasis of prostate cancer cells induced by the AR [80]. The influence of peptide hormones on the onset and progression of prostate cancer is significant. Notably, 6PPDQ appears to mediate prostate carcinogenesis by interacting with these hormonal pathways. This interplay suggests potential avenues for therapeutic intervention and further research into the mechanisms underlying hormone-driven prostate cancer development.

Membrane rafts are dynamic domains of lipid accumulation on the cell membrane, which play an important role in cell signal transduction pathways, particularly in the proliferation and metastasis of cancer cells [81]. EGFR, located in membrane rafts, mediates the PI3K/AKT signaling pathway, thereby promoting the survival of prostate cancer tissues. Meanwhile, the level of membrane cholesterol plays a crucial role in maintaining the stability of these lipid rafts, and by controlling cholesterol levels, the destruction of lipid rafts can be induced, leading to the apoptosis of cancer cells [82,83,84]. These specialized microdomains within the cell membrane facilitate various signaling pathways that support tumor progression and resilience, indicating their importance in the survival of prostate cancer. In addition, membrane rafts also function in the invasion of prostate cancer. The signal transduction in which IL-6 and STAT3 participate may influence cell invasiveness through the AR, and this has been shown to depend, at least in part, on lipid raft function [85]. In addition to membrane rafts, Caveolae, serving as a special concave domain on the cell membrane, are involved in cell transport and signal transduction [86]. Caveolin-1 is a critical component of caveola formation. The abnormal expression of caveolin-1 affects the typical performance of caveola function, and its expression level is of great significance to caveolae [81]. Moreover, it has been confirmed that caveolin-1 is abnormally expressed in prostate cancer cells and is related to the progression of prostate cancer [87,88,89].

The androgen axis and its nuclear receptor, the androgen receptor (AR), play a pivotal role in the carcinogenesis of prostate cancer and the progression of the disease. AR functions as a ligand-activated transcription factor, with its activity modulated by hormonal levels and coactivator availability [90]. Furthermore, the overexpression of the AR can quantitatively enhance its sensitivity to androgens, resulting in alterations to its physiological roles. This overexpression leads to an increased number of binding sites that facilitate the expression of cell-cycle regulators, thereby promoting the proliferation of cancer cells in castration-resistant prostate cancer [91]. In addition, more NR also influences prostate cancer. In the retinoic acid receptor family, the overexpression of RARβ has been found to reduce the proliferation of prostate cancer cells [92], and RARγ may inhibit the development of prostate cancer by competitively binding AR binding sites [93]. The expression of retinoic-acid-receptor-related orphan receptor (RORγ) is significantly upregulated in prostate cancer, which subsequently drives the expression of the androgen receptor (AR). The antagonism of RORγ can effectively inhibit AR expression and impede prostate cancer growth, underscoring the critical role of stable RORγ expression in prostate cancer progression [94,95]. In previous studies, it has been shown that the expression level of RXRα in prostate cancer is diminished compared to in non-malignant prostate tissue. However, the overexpression of RXRα results in the proliferation of prostate cancer cells decreasing and inducing apoptosis [96].

Regarding transmembrane protein kinase activity, receptor tyrosine kinases (RTKs) are a diverse family of transmembrane proteins that can activate multiple pathways upon receptor binding. RET is a transmembrane protein tyrosine kinase characterized by proto-oncogenic properties, and it has been identified as a potential promoter of the invasive capabilities of prostate cancer through the activation of p70S6 kinase [97]. Furthermore, the dysregulation of various other RTKs has been implicated in numerous cancer types [98].

### 4.4. Molecular Docking Between 6PPDQ and Core Proteins

Finally, the molecular docking method verified the interaction between 6PPDQ and potential proteins. The results showed that the binding energy values for the interaction between 6PPDQ and these essential proteins were between −6.1 and −8.8 kcal/mol. According to the Vina scoring mechanism, lower binding energy represented better intermolecular affinity and a higher level of molecular interaction, suggesting highly stable binding between all ten proteins and 6PPDQ.

Network toxicology, based on traditional network pharmacology, has emerged as a valuable tool for investigating potential toxicity. The integration of network toxicology with molecular docking has the potential to enhance the predictive capabilities regarding environmental toxins and their implications for human health.

However, we still have some limits. Firstly, some related targets of 6PPDQ may need to be addressed due to insurmountable challenges in obtaining processes in databases. Furthermore, although multiple signaling pathways of prostate cancer were proven to be targeted by 6PPDQ in GO and KEGG enrichment analysis, these specific mechanisms are still required for further investigation. Finally, our study provides a foundation for future research. It stresses the latent harmful substance, but for further understanding of the relation between 6PPDQ and prostate cancer, more epidemiological and clinical studies are needed.

## 5. Conclusions

Our findings indicate that 6PPDQ influences numerous cancer-associated proteins, potentially elevating the risk of cellular transformation into malignancy by disrupting the normal physiological functions of these biomolecules. The underlying mechanism of molecule action and latent signaling pathways of 6PPDQ-related targets relevant to cellular carcinogenic pathways suggest the hypothesis of its potential carcinogenicity.

In conclusion, our study supports a new perspective on evaluating the correlation between chemical product contamination and human tumorigenesis. This finding may influence future diagnostic strategies for prostate cancer. We need to devote more attention to chemical substance pollution and cancer and more investigations into the signaling pathways of how 6PPDQ contributes to prostate cancer.

## Figures and Tables

**Table 1:** The core target information of PPI network.

Receptors	Degree	Average Shortest Pathlength	Betweenness Centrality	Closeness Centrality	Neighborhood Connectivity	Topological Coefficient
SRC	38	2.288888889	0.167929308	0.436893204	13.02631579	0.117354196
PIK3R1	27	2.544444444	0.028902605	0.3930131	13.96296296	0.164270153
EGFR	26	2.355555556	0.057176398	0.424528302	16	0.148148148
GRB2	25	2.627777778	0.056225201	0.380549683	12.96	0.161
PTPN11	21	2.711111111	0.011173159	0.368852459	14.33333333	0.188596491
IRS1	18	2.55	0.01546598	0.392156863	18.5	0.203296703
JAK2	18	2.777777778	0.017949836	0.36	15.05555556	0.217391304
KDR	15	2.688888889	0.030385955	0.371900826	14.73333333	0.187179487
IGF1R	13	2.727777778	0.006617419	0.366598778	19.61538462	0.254745255
SOS1	11	2.894444444	0.004922684	0.345489443	16.81818182	0.274217586

**Table 2:** The binding energy of 6PPDQ and core targets (kcal/mol).

Receptors	PDB ID	Vina Score	Cavity Size	Center			Size		
				X	Y	Z	X	Y	Z
JAK2	6e2q	−8.8	1731	251	−45	59	22	22	22
SRC	2h8h	−8.7	699	18	25	57	22	22	22
GRB2	1gri	−8.7	865	29	74	22	22	22	22
PTPN11	7vxg	−8.5	914	41	29	44	22	22	22
IGFR1	6jk8	−8.4	1956	112	104	155	22	22	22
EGFR	7syd	−8	10,952	162	172	164	22	35	35
IRS1	1qqg	−7.9	1317	12	50	14	22	22	22
PIK3R1	2v1y	−6.5	168	73	44	−25	22	22	35
SOS1	3ksy	−7.6	1849	45	43	69	31	31	22
KDR	1vr2	−6.7	539	35	32	17	22	22	22

## Data Availability

The authors confirm that all data underlying the findings are fully available without restriction. All relevant data are within the paper.

## Supplementary Materials

The following supporting information can be downloaded at: https://www.mdpi.com/article/10.3390/toxics12120891/s1, Table S1: top five of ranked skeleton with Vina score.
