# Supplementary figures and images for: Investigating the Potential Effects of 6PPDQ on Prostate Cancer Through Network Toxicology and Molecular Docking
Source: Toxics. 2024 Dec 8;12(12):891. doi: 10.3390/toxics12120891 (PMC11728691; doi:10.3390/toxics12120891)

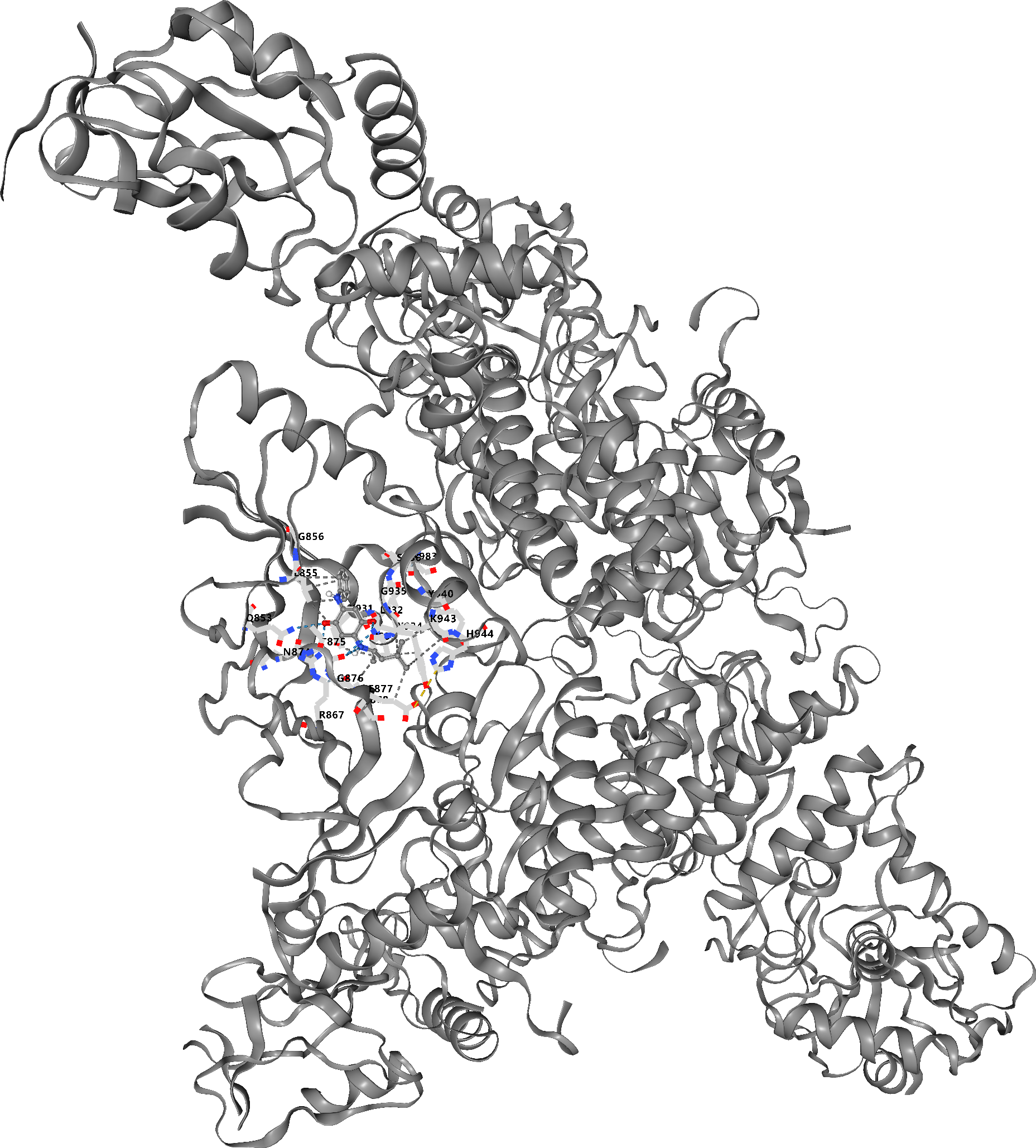

Supplement: Supplementary file 1 [file toxics-12-00891-s001.zip › 4gl9 dock.png]

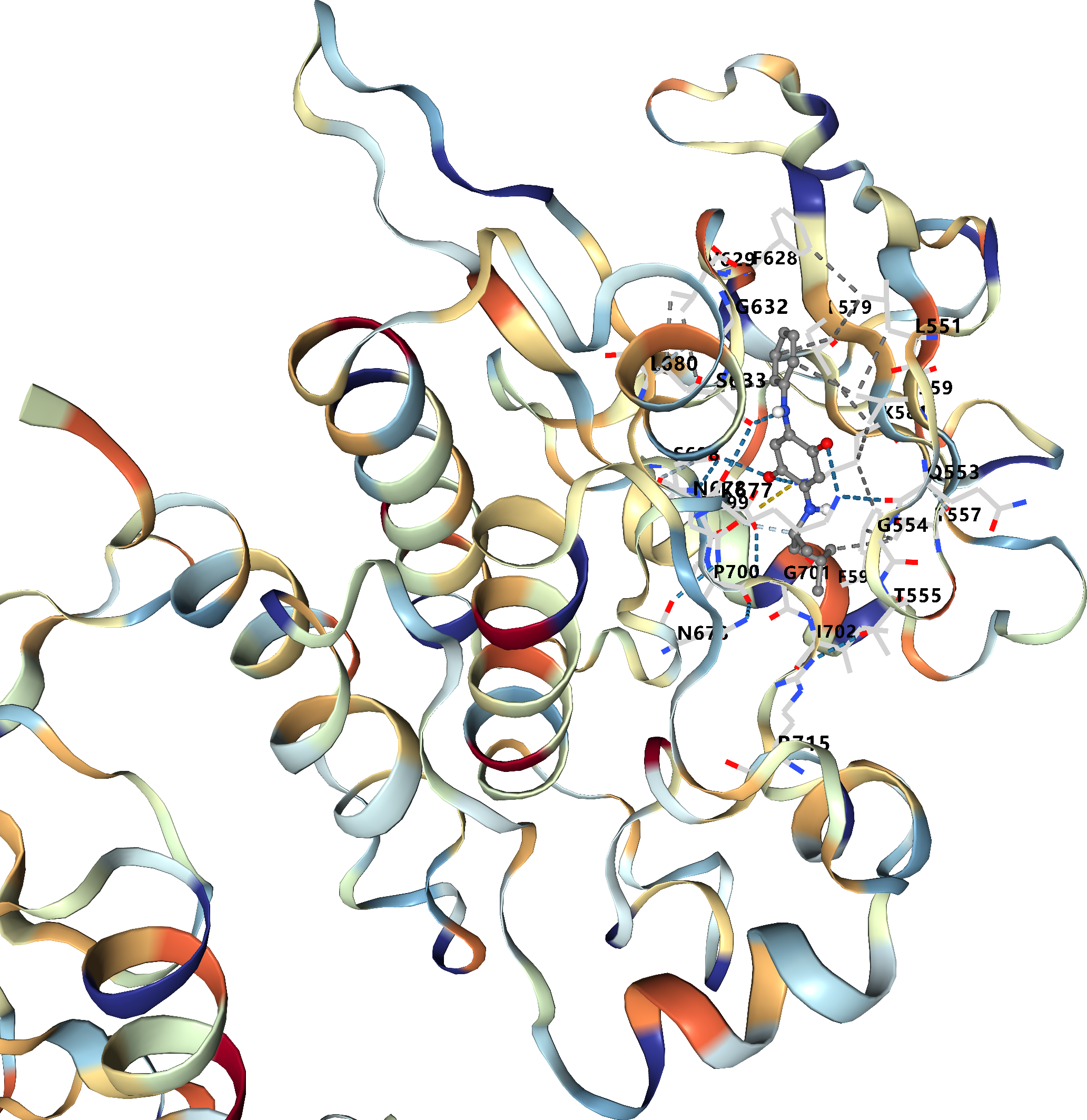

Supplement: Supplementary file 1 [file toxics-12-00891-s001.zip › 7f7w dock.png]
